# Unsupervised and interpretable discrimination of lithium-bearing minerals with Raman spectroscopy imaging

**DOI:** 10.1016/j.heliyon.2024.e35632

**Published:** 2024-08-03

**Authors:** Diana Guimarães, Catarina Monteiro, Joana Teixeira, Tomás Lopes, Diana Capela, Filipa Dias, Alexandre Lima, Pedro A.S. Jorge, Nuno A. Silva

**Affiliations:** a Center for Applied Photonics, INESC TEC, Rua do Campo Alegre 687, Porto, 4169-007, Portugal; b Departamento de Física e Astrofísica, Faculdade de Ciências da Universidade do Porto, Rua do Campo Alegre 687, Porto, 4169-007, Portugal; c Departamento de Geociências, Ambiente e Ordenamento do Território, Faculdade de Ciências da Universidade do Porto, Rua do Campo Alegre 687, Porto, 4169-007, Portugal; d Instituto de Ciências da Terra, ICT, Polo da Universidade do Porto, Porto, 4169-007, Portugal

**Keywords:** Spectral imaging, Interactive visualization, Photogrammetry, Lithium minerals, Raman spectroscopy, Raman imaging

## Abstract

As lithium-bearing minerals become critical raw materials for the field of energy storage and advanced technologies, the development of tools to accurately identify and differentiate these minerals is becoming essential for efficient resource exploration, mining, and processing. Conventional methods for identifying ore minerals often depend on the subjective observation skills of experts, which can lead to errors, or on expensive and time-consuming techniques such as Inductively Coupled Plasma Mass Spectrometry (ICP-MS) or Optical Emission Spectroscopy (ICP-OES). More recently, Raman Spectroscopy (RS) has emerged as a powerful tool for characterizing and identifying minerals due to its ability to provide detailed molecular information. This technique excels in scenarios where minerals have similar elemental content, such as petalite and spodumene, by offering distinct vibrational information that allows for clear differentiation between such minerals. Considering this case study and its particular relevance to the lithium-mining industry, this manuscript reports the development of an unsupervised methodology for lithium-mineral identification based on Raman Imaging. The deployed machine-learning solution provides accurate and interpretable results using the specific bands expected for each mineral. Furthermore, its robustness is tested with additional blind samples, providing insights into the unique spectral signatures and analytical features that enable reliable mineral identification.

## Graphical abstract

**Figure:**
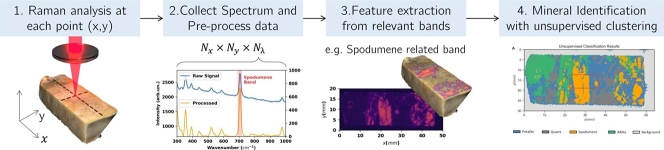

## 1 Introduction

Lithium (Li) is a vital component in energy storage systems and rechargeable batteries and has been recently added to the 2020 EU Critical Raw Materials list based largely on its importance in green technologies [1]. Li plays a crucial role in powering contemporary technologies and, particularly, in promoting eco-friendly transportation. The latter significantly increased the demand for Li in recent years, making the location and characterization of Li sources and deposits a problem of uttermost importance [2].

In a simplified global picture, the major sources of Li are brine deposits and hard rock pegmatites. Featuring non-trivial advantages, such as faster production cycles, diversification of supply locations, and proximity to end markets, pegmatite sources are becoming increasingly appealing [3]. In short, pegmatites consist of igneous rocks enriched in rare elements and with relatively coarse grain-sized crystals favoring the extraction process. Examples of Li pegmatite minerals include spodumene, petalite, and lepidolite, with spodumene standing out as the primary supplier of Li due to its abundant deposits, higher Li content, and more efficient methods of processing [4]. As Li content may vary from mineral to mineral, the correct identification of mineral type is a critical part of the mining industry, with impact in stages from prospection to exploitation and even processing. For example, taking into consideration spodumene and petalite - a common scenario in multiple European hard-rock deposits [5], [6] - spodumene features a theoretical concentration at 3.73% while petalite stands at 2.09% [7]. Furthermore, the simultaneous presence of both minerals in fine-grain mixtures poses a challenge to their identification, as they may appear homogeneous to the naked eye [8]. This may strongly influence the estimation of potential Li resources, the planning of mining operations, the choice of processing methods, and the suitability for different applications. In this context, developing tools for unbiased identification of Li-bearing minerals is a topic of paramount importance and impact within the mining industry.

While benchmark methods such as Laser Ablation Inductively Coupled Plasma Mass Spectrometry (ICP-MS), or X-ray Diffraction (XRD), can provide reliable mineral identification, their high cost, complex sample preparation, demanding operator skills, and time-consuming tasks often hinder their potential [9], [10]. Closer to industrial environments by featuring significantly minimal sample preparation and shorter analysis time, techniques such as Laser-Induced Breakdown Spectroscopy (LIBS), have been proving their potential for mineral identification in a wide range of application fields [11], [12], [13]. However, LIBS-based solutions may struggle to deliver good performances for more complex problems, in specific when minerals share the same chemical elements and in the presence of fine-grain mixtures - such is the case of petalite and spodumene.

Although less used in industry due to the higher cost of the spectrometer, less-interpretable information, and lower throughput, Raman spectroscopy (RS) provides an interesting alternative in terms of mineral identification. Raman Spectroscopy uses a laser to excite vibrational modes of the crystal lattice, thus holding the potential to identify minerals solely based on the obtained scattering signature [14]. In short, when a monochromatic laser beam hits a spot (typically focused), it interacts with the sample and most of the photons suffer elastic scattering (Rayleigh scattering). Yet, a small fraction can be scattered in an inelastic process, due to the interaction with the vibrational modes of the molecules existing in the analyzed spot, losing its energy. This energy difference can be noticed by analyzing the wavelength spectrum of the Raman scattering signal, which contains information about the sample molecular structure and can be related to the mineral type [14].

Focusing on the specific case of Li mineral identification, Raman spectroscopy presents non-trivial advantages when compared to reference techniques such as ICP-MS and ICP-OES. On one hand, because RS is non-destructive and provides information based on the characteristic mineral vibrational bands, it is particularly valuable when distinguishing Li-minerals that may coexist and have similar chemical compositions, only differing slightly in elemental concentration such as the case of spodumene and petalite. Indeed, these small differences and valuable spatial and mineral crystallinity information can be easily lost after sample digestion that is required for analysis by these reference techniques. On the other hand, RS can also provide spatial analysis by scanning the sample surface and constructing informative spectral maps. This technique, referred to as Raman imaging, is an extremely helpful tool to analyze heterogeneous samples, providing information regarding the molecular distribution within a sample [15], [16] and can find multiple applications at the technology level.

Overall, in the context of mineral identification, RS has been used in a wide span of contexts including archaeometry [17], [18] and extreme environments such as the Artic sea [19] or volcanic eruptions [20], just to name a few examples. It has also been widely used in the jewelry context for gemstone identification [21], [22], and even on extraterrestrial targets [23], [24]. One of the hallmarks of the RS potential in minerals studies is the RRUFF database, which provides an extensive dataset of Raman signatures for almost all mineral species known to date and that have been collected by geologists since 2006 [25]. Focusing on Raman imaging, the technique can be relevant not only for academic purposes, such as understanding the distribution of phases or interaction of distinct minerals [26], [27] but also for mining-related applications, such as providing estimates of the mineral content of samples at a given mining site [28], [29].

However, RS also has its drawbacks when it comes to mining-related applications. On one hand, from the side of the technique itself, the high fluorescence background [30] and shot noise can hinder the extraction of the sample vibrational information [14]. On the other hand, even if a good signal can be extracted, the task of correctly pinpointing the mineral composition can be extremely complex due to the chemical compositions and properties of minerals. Indeed, a considerable number of minerals have very little difference in molecular design, meaning that their Raman spectra are quite similar. In this context, machine learning and data-driven solutions have been extensively explored in the literature, in particular exploiting the benefits of supervised learning. In the typical scenario, a data-driven classification model is trained with a specific dataset with associated mineral type labels (for example using the RRUF database), before being ready to apply to blind samples. Examples of such algorithms include approaches with support-vector machines, Partial-Least Squares, k-nearest neighbors, random forests, ANNs, and more recently Deep and Convolutional Neural Networks [31], [32], [30], [33], [34]. Nonetheless, some of these solutions often have a black-box character, such as ANN that handles input data in a non-linear manner through numerous layers of interconnected nodes and a large number of parameters, being challenging to understand. Others are based on distance metrics between a specific reference dataset and the experimental data which can be prone to errors (e.g. instrumental drifts), hindering the interpretability and performance of the solution and impairing the end-user experience.

**Figure 1:**
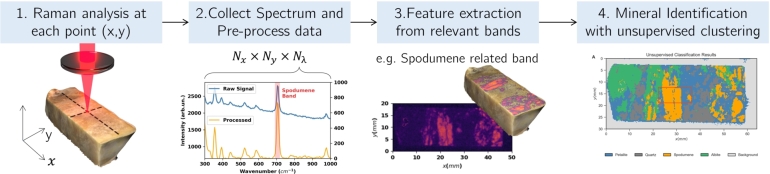
Conceptual illustration of this study workflow for the deployment of a Raman imaging algorithm to perform unsupervised mineral discrimination and identification.

In this work, a Raman imaging solution is explored for the automatic identification of minerals using an unsupervised clustering methodology (see Fig. 1 for illustration purposes). More specifically, it is proposed a processing pipeline that starts with a feature extraction involving relevant bands for the minerals of interest, followed by a k-means clustering and hyperparameter optimization procedure. Using informed cluster initialization together with radar charts to provide an additional degree of interpretability, this methodology is applied to a case study involving the differentiation of spodumene and petalite. Both for a training sample and blind test samples, it is shown that the solution developed provides reliable identification of minerals at the surface of the sample and thus may open a window of opportunity to explore the use of such RS solutions for Li-mining applications for industrial and *in-situ* environments.

## 2 Materials and methods

In this work, the use of RS and Raman Imaging is explored for the discrimination of Li-bearing minerals. More specifically, the primary goal is the discrimination between petalite and spodumene, which constitutes a major challenge for other techniques due to their similar chemical formula but that is of high relevance for prospective mining sites in Europe [5], [6]. Furthermore, the focus will also be put on the deployment of a solution that is able to perform in relevant industrial contexts, i.e. capable of warranting robustness and high throughput, meaning lower integration times and possibly lower signal-to-noise ratios.

### 2.1 Samples description

The samples used in this work comprised three rock samples with a mineralogical composition that included petalite, spodumene, albite, and quartz, originating from the same aplite-pegmatite located in Vila Pouca de Aguiar, Portugal. They were obtained from an “Instituto Geologico e Mineiro” drilling campaign, where prospection drillings were made in the Adagói pegmatite from the Barroso-Alvão field [35]. The samples were collected specifically from the “ADG-2” drilling. The core was cut using a diamond saw blade to obtain a planar cross-section, and kept in the warehouse of “Laboratório de Energia e Geologia” - campus of S. Mamede de Infesta. The rock slab was also used to produce a thin section that was observed in a Nikon OPTIPHOT-POL polarizing microscope coupled to a cold-cathodoluminescence equipment (CITL CCL 8200 MK4) to learn the mineralogy of the sample. This flat surface will improve the quality of the Raman measurement and further mineralogical analysis, reducing the effects of height differences between samples. However, no polishing procedure was performed, which means that some porosity and roughness may still introduce some minor signal variation.

### 2.2 Raman imaging system

**Figure 2:**
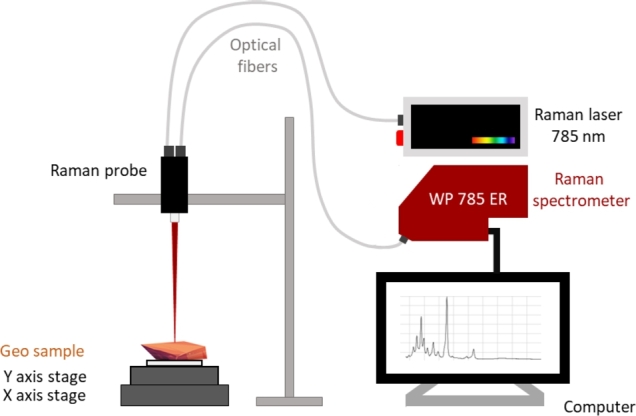
Schematics of the Raman imaging system.

The Raman imaging system used in this study consists of an excitation laser diode (Coherent SureLock BT-785) with a central wavelength of 785 nm and an output power set to 200 mW to avoid sample degradation. Spectra were acquired at each scanning point using a spectrometer (Wasatch Photonics WP 785 ER) with a spectral range spanning from 270 to 3350 cm^−1^ and a typical spectral resolution of 8 cm^−1^. The data was acquired with an integration time of 200 ms, from a single sample. The laser and spectrometer were fiber-coupled to a Raman probe tip (Ballprobe RP 785 from Wasatch Photonics) with a spot size of 200 μm, as illustrated schematically in Fig. 2. The probe was placed at the focal distance from the sample, which corresponds to approximately 5 cm.

To observe the spatial distribution of Li minerals in the pegmatite sample, the samples were placed on an XY motorized translational stage, and the spectra were acquired by performing a whiskbroom scanning with a step size of 200 μm. In total, acquiring the representative spectrum of the pegmatite sample took around 3 hours.

### 2.3 Pipeline for the mineral identification algorithm

When organizing the methods used in the literature regarding the data fed to the machine learning model, two large groups are found: i) methods based on the extraction of features from specific bands; and ii) methods that use the entire spectral range. As finding a set of exclusive bands for thousands of minerals is virtually impossible, the latter may, in theory, be promising for the deployment of a universal mineral classifier [30]. Nonetheless, being based on distance metrics such as cosine similarity [32], it highly relies on the quality of the signal obtained (meaning higher integration times and thus lower throughput) and requires training procedures with some reference samples with the same instrument. Besides, a robust industrial tool would definitely need some regular calibration procedures to avoid biasing the analysis due to environmental conditions or instrumental drifts.

Taking into account the cost, lower throughput, and accessibility issues of such an approach, a methodology based on the extraction of specific peaks seems more reasonable for mining-related applications. Indeed, provided the number of mineral types to be discriminated is small and relatively stable (e.g. as in a mining site), one can select a relatively reduced set of features to be extracted to accomplish a good qualitative analysis by focusing on mutually exclusive Raman scattering bands. In such a context, substituting a one-fits-all with a fit-for-purpose approach, with a lower computational footprint together with an interpretable framework, can be extremely beneficial for the end-user, as target-specific performance and interpretability are more important than having a broader knowledge that includes less common minerals.

For the purpose of this manuscript it was used an approach based on unsupervised clustering, avoiding the necessity of labeled training samples (either reference or synthetically generated ones) which may ease the training process. Besides, putting into the end user perspective, when combined with interpretable tools the unsupervised approach may provide a good setting to tackle a wide range of problems while still providing the necessary feedback for informed decisions, warranting a higher degree of control of the results' quality, robustness, and versatility [36].

**Figure 3:**
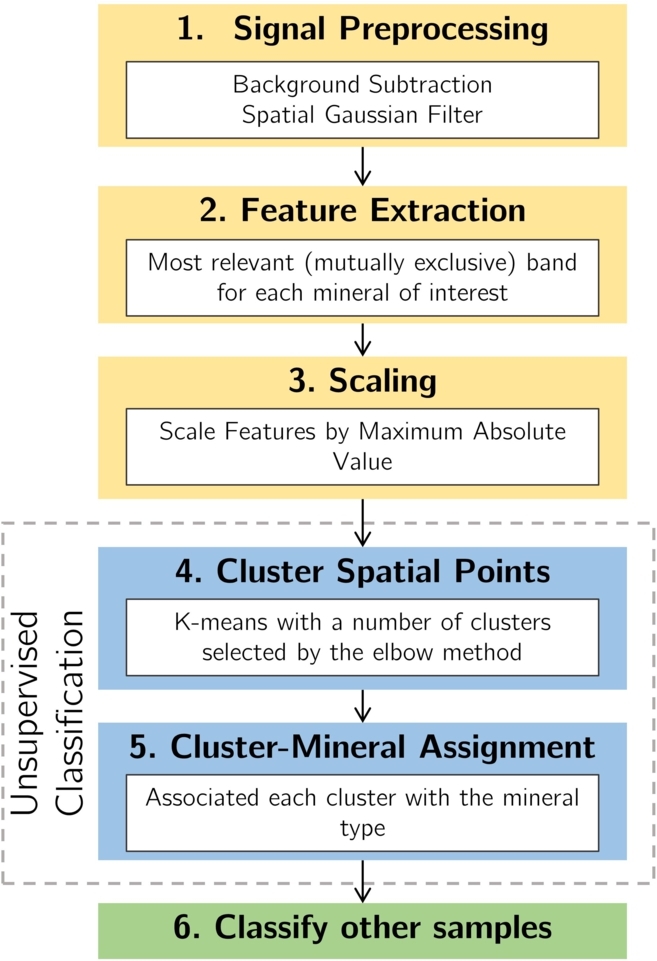
Processing pipeline utilized for mineral discrimination and identification.

All the pipeline and processing workflow was deployed as a standalone computer algorithm written in Python, using standard processing (*scipy* [37]) and machine learning (*scikit-learn* [38]) libraries. The processing pipeline of the classification method deployed involves the sequential steps (see Fig. 3):

**1. Preprocessing:** The information acquired in Raman spectroscopy often contains a continuous fluorescence component that is not related to the molecular structure of the mineral itself and that may hide specific information of the molecular bands. Therefore, in order to mitigate these effects, each acquired spectrum underwent a preprocessing stage starting with a standard Asymmetric Least Squares (ALS) smoothing algorithm to remove this influence. In addition, to take into account possible variations of the acquired intensity due to surface sample properties (e.g. roughness, cleanliness) each spectrum was normalized to the sum of the total intensity. Finally, a Gaussian filter was applied in the spatial domain to account for possible outliers.

**2. Feature extraction:** In order to deploy a robust solution, the cornerstone of this algorithm will be the extraction of relevant features corresponding to exclusive bands of the minerals of interest. These bands were selected according to two criteria: first, the most intense bands of the Raman spectra were located, based on the RRUF mineral database for 785 nm of excitation wavelength; then, it was verified that these had no interferents in the given group of minerals. Note that while, for simplicity, we chose to use independent emission bands, the same approach can be applied to spectra where there is interference between the existing minerals. Selecting the relevant bands, for each pixel and from each spectrum, it was extracted a total of $N_{bands}$ corresponding to a dimensionality reduction of the total hyperspectral cube $N_{x}\times N_{y}\times N_{\lambda }$ into a smaller dataset of dimension $N_{x}\times N_{y}\times N_{bands}$.

**3. Data Scaling:** The next step in the workflow is data scaling, which is performed in order to further mitigate the effects of Raman signal variation due to surface properties and to avoid any biasing of the model due to the relative strength of bands. In a simplified picture, this allows us to effectively translate mineral identification into a qualitative problem, enhancing the robustness of the methodology while also easing transfer learning between distinct systems. For this algorithm, the *MaxAbs Scaler* of the *scikit-learn* library was used, scaling each feature to its maximum value.

**4. Clustering:** With the dataset reduced and scaled, the next step is to proceed with the training of a data-driven model to perform mineral-type discrimination. In particular, and following the arguments discussed in previous sections, it was selected an unsupervised approach using a k-means clustering model from the *scikit-learn* [38] library. To correctly optimize the number of clusters $N_{c}$, one can follow the empirical elbow criteria [36] together with the direct comparison to the number of minerals to identify. For each sample, and after using one or multiple samples to train the algorithm, the unsupervised clustering labels each pixel of the dataset into the set of $N_{c}$ clusters by updating the position of $N_{c}$ centroids iteratively until convergence.

**5. Interpretable Assignment:** Using an unsupervised methodology allows the discrimination of the spatial points into clusters but does not associate them directly with the mineral type. For this step, and aligned with a previous suggestion for LIBS [36], it is proposed the use of a semi-quantitative methodology that relates feature relevance and mineral type. Furthermore, in the case where mutual exclusive bands can be found, one can also initialize the centroids of the clusters close to the expected results, thus easing the process of mineral-clustering assignment.

**6. Classification of blind samples:** Applying the same preprocessing and scaling procedures, a pre-trained clustering model can then be used to accurately classify minerals on test samples, provided these share similar mineralogical constitution.

## 3 Results

To infer the capabilities and validate this methodology, it was investigated a case study concerning the identification and distinction of two Li-bearing minerals, coexisting in the same sample and having a heterogeneous surface distribution. A set of 3 different samples of similar mineral content were used, retrieved from the same aplite-pegmatite aggregate. In this way, it is possible to demonstrate the capability of using the methodology proposed for the discrimination of relevant Li-bearing minerals while also paving for real-world applications by proving the generalization capabilities for unseen samples in a given mining exploration site.

### 3.1 Raman analysis of the mineral samples

The typical chemical formula of the minerals present in the samples can be found in Table 1. As can be observed, distinguishing the minerals in terms of the presence or absence of specific elements is not trivial as petalite and spodumene contain exactly the same elements.

**Table 1:** Chemical formula and relevant Raman bands of the minerals observed on the sample.

Mineral	Chemical Composition	Relevant Band
Albite	NaAlSi_3_O_8_	509 cm^−1^; 479 cm^−1^
Petalite	LiAlSi_4_O_10_	490 cm^−1^
Spodumene	LiAlSi_2_O_6_	705 cm^−1^
Quartz	SiO_2_	464 cm^−1^

In Table 1, the most relevant Raman bands can also be found. The characteristic bands for Albite are located at 509 and 479 cm^−1^ and are linked to the O-Si-O and O-Al-O bending and stretching vibrations of the four-membered tetrahedral rings [39]. Natural petalite shows a prominent Raman band at 490 cm^−1^, originating from the symmetric bending vibrations of the silicate tetrahedrons SiO_4_. The satellite of this band can be seen at 467 cm^−1^ [40]. Concerning spodumene, the more significative Raman band is located at 705 cm^−1^ and is a consequence of the bridging stretching modes of Si-O [41]. For quartz, the dominant Raman band is at 464 cm^−1^. It represents a characteristic vibrational mode associated with the Si-O-Si bending within the quartz crystal lattice, and it is the band least affected by the crystallographic orientation [42].

**Figure 4:**
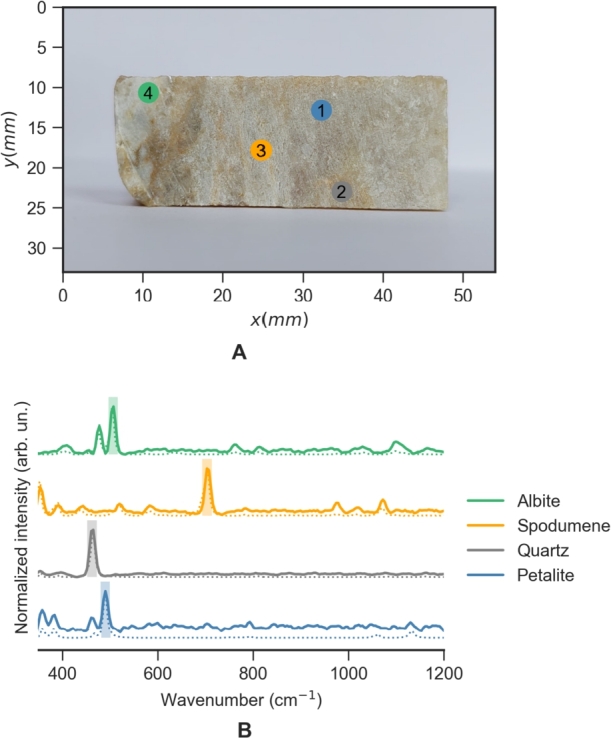
A) Photograph of the training sample with the regions of the main minerals annotated with colored circles B) Raman spectrum acquired on the spots of the corresponding color (full line) and the corresponding reference spectra retrieved from the RUFF database: Albite - R100169; Spodumene - R060039; Quartz - R150074; Petalite: R060365 (dot lines).

As can be seen, these bands are located in different areas of the spectrum allowing differentiation of the minerals based on their absence/presence in each spatial point. In fact, the sample chosen for the training set has both petalite and spodumene in well-defined spatial regions which can ease the analysis of the results. A total study area of 5x2 cm, which included the background (Teflon) and the pegmatite sample of 4.5 x 1.5 cm (Fig. 4A) was used to train the algorithm, which corresponds to 25000 spectra. The measurement step size was 200 μm. In Fig. 4A) it is also shown the 4 distinct spots where the main 4 minerals are known to be present. Due to the size of the crystals, these regions could be easily identified by the naked eye. However, this might not be always possible, particularly when the size of the crystals is smaller and minerals can be simultaneously present in fine-grain mixtures hindering their identification. Indeed, while the spodumene crystals are relatively large in this sample, some regions of petalite are known to be embedded in quartz matrix, which one needs to account for a proper analysis.

The acquired spectra obtained for each mineral spot are shown in Fig. 4B). The presented spectra are the result of the average signal concerning a grid of 5 x 5 points (area of 1 mm^2^) neighboring the selected point. These results are in accordance with the RUFF database spectra selected for these minerals as can be seen by the dotted lines in the graphics. The spectra were chosen based on the similarity between the data base mineral and this case study in terms of color and texture (if polished or raw, i.e. in its natural state); and also based on the laser wavelength used selecting spectra with a wavelength close to 785 nm. Note however that the petalite zone shows a prominent band in the region of 464 cm^−1^ which may be related with quartz mineral as mentioned before.

**Figure 5:**
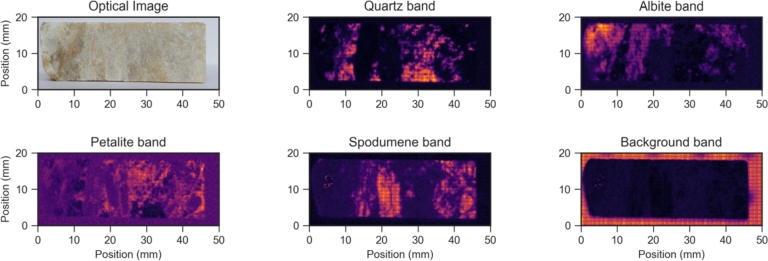
Photograph of the sample and the corresponding Raman maps for each characteristic mineral band present on the sample.

Emphasis is also given to the most intense Raman band in order to aid the visual analysis. Taking these mineral reference Raman bands into account, a map can be created for the whole sample region (see Fig. 5). An extra region called Background, that represents the Teflon sample holder, is also depicted and will also be handled by the training algorithm for robustness purposes. It is possible to notice that in general, the minerals have distinct and exclusive spatial distributions on the sample area and none can be found homogeneously across all the analyzed surface, which strongly support the methodology chosen. Indeed, analyzing these spatially resolved Raman data it is possible to state that, for this type of sample, the characteristic Raman band intensity even with such small integration times is enough to construct and train a machine learning algorithm that will automatically identify different mineral regions. Still, one shall observe that petalite distribution is not as clear as the others and may superimpose with other minerals such as quartz. While this was in part expected for this sample as previously discussed, it is also important to note that the effect may be more pronounced due to the fact that the associated Raman band to petalite features a lower signal-to-noise ratio.

### 3.2 Training and interpretability

**Figure 6:**
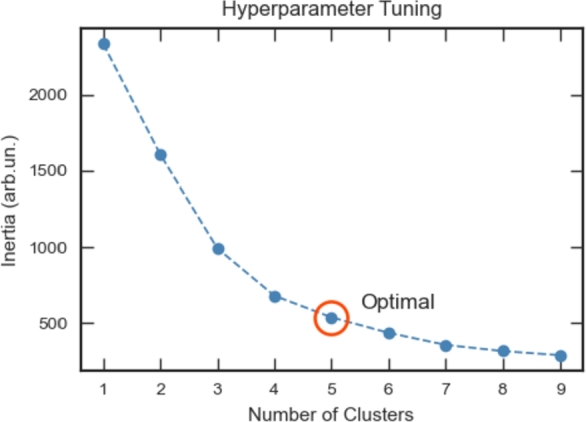
Total cluster inertia as a function of the number of clusters selected for K-means when applied to the training sample.

As previously referred, the proposed methodology involves extracting the characteristic band lines for each mineral, that are stored as features for each pixel. After this extraction, each feature is normalized by its maximum absolute value before applying the K-means clustering algorithm. Although K-means is an unsupervised methodology, the optimal number of clusters, $N_{c}$, still needs to be chosen in order to fine tune the algorithm and provide accurate results. This hyperparameter tuning procedure is commonly task-specific and should be interpreted taking into consideration the study context. For simplicity, it was used the empirical *elbow method* [43], [36], that represents the total inertia as a function of the number of clusters and seeks the elbow point, where there is a good compromise between minimizing intra-cluster distance and the number of clusters. From Fig. 6, the elbow point appears to be between 5 and 6. Taking into consideration the context of the problem, it is expected to observe 5 clusters (4 mineral types plus the background cluster), and thus 5 is taken as the optimal $N_{c}$.

**Figure 7:**
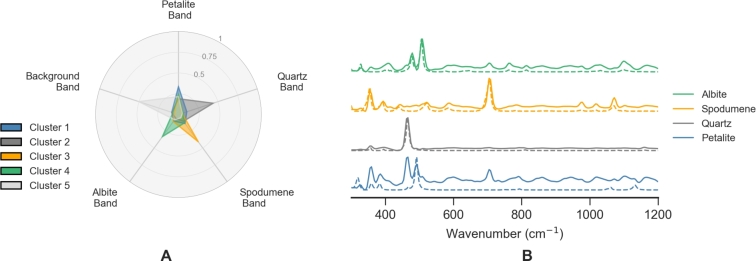
A. Radar charts to assign the correct mineral label to each cluster. B. Each cluster is represented by the mean Raman spectrum, together with the label assigned.

Having the number of clusters identified, the next step is to correctly assign each cluster to a mineral label. To do this it was developed a visual tool, using radar charts, that is depicted in Fig. 7. In Fig. 7A, the mean value of the Raman characteristic bands for each mineral is represented for each cluster centroid (in distinct colors). In this way, it is easy to link the cluster to the mineral by looking at the highest contribution of the several characteristic bands. For example, the centroid of cluster 3 has high values for the spodumene band being automatically assigned to the spodumene mineral. It is interesting to observe that in some cases like petalite, shown more clearly in Fig. 7B, characteristic bands of other minerals, like quartz and spodumene, are also present on the mean spectrum of the cluster centroid. Therefore, the petalite cluster spectrum will be different from the pure petalite mineral spectrum retrieved from the RRUF database [25] (dashed line). This might be explained due to the fine grain mixtures, or even due to the fact that, depending on their crystal structure, different minerals will have more intense Raman signals. In fact, it is known that well-ordered highly symmetrical structures, like quartz or spodumene, give a more intense Raman signal [14]. The petalite signal will have a lower signal-to-noise ratio and even small (spatially or in terms of concentration) contributions from other minerals might be enough to deem an appearance in the petalite cluster spectrum.

It should be emphasized that, in order to prevent cluster and label changes with each run of the procedure, a fixed random seed should be selected for the k-means algorithm, or it should be initialized with cluster centroid guesses based on the expected mineral characteristic composition. This approach ensures the consistency of the results for the same dataset.

**Figure 8:**
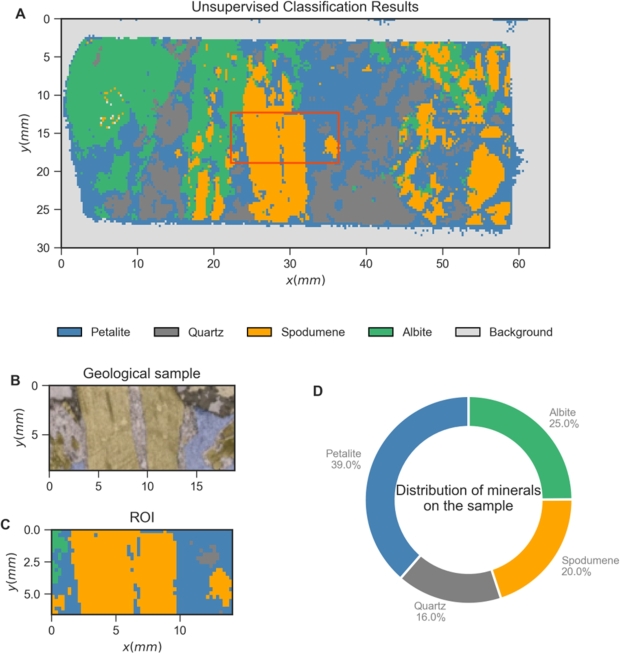
Results obtained for the training sample after using the complete algorithm. (A) Unsupervised Raman cluster results with identified clusters assigned to minerals; (B) Petrographic analysis of part of the sample where the area in yellow represents spodumene and the blue part represents petalite; (C) Detail of map (A) corresponding to the area on the image (B); (D) Results representing the amount of mineral present on the training sample.

With the clusters assigned to each mineral type, a map depicting the mineral type distribution can be constructed as shown in Fig. 8). By comparing the cluster map (8A) with the expected mineral regions presented in the petrographic image obtained for a smaller region of the sample (8B), it is visible that the results are in good qualitative agreement. Finally, one can also construct a pie chart as in Fig. 8D, providing an interpretable and relevant metric and analysis tool to show how much of each mineral is present in the sample. Such metric may allow the end-user to quickly infer about the Li content of that sample and, in an industrial environment, to make informed investment decisions.

### 3.3 Testing in blind samples

**Figure 9:**
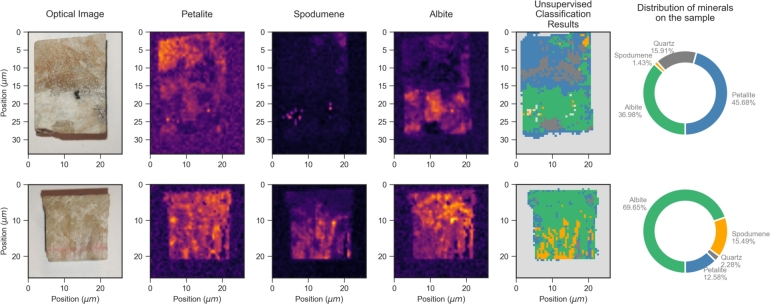
Results of the trained clustering algorithm for blind test samples. (Left) Sample photograph; (Center) Raman maps for each major mineral present on the sample - petalite, spodumene and albite (Right) Unsupervised Raman cluster results based on Raman mapping; (Far right) Percentage distribution of minerals present on the sample.

Finally, in order to validate the automatic mineral identification strategy based on Raman imaging, the previously trained model was applied to two distinct unknown test samples having similar mineralogy obtained from the same site. Upon analyzing the results presented in Fig. 9, a clear correlation between the expected mineral distribution (observed in the accompanying maps) and the classification algorithm is again evident for spodumene and albite minerals. In what concerns the distribution of petalite, in particular for the sample in the bottom line of Fig. 9, the analysis is less clear due to the lower intensity of the petalite Raman scattering signal and the fact that the relevant band lays close to the albite one. Yet, even in this case, the presence of a strong albite-related feature still enables the minerals to be correctly identified. Overall, these results demonstrate that the methodology successfully fulfills its intended purposes, suggesting that with appropriate training samples and signal analysis, this approach can function as an efficient and robust mineral identification tool. The estimated analysis time for a sample, including both acquisition and processing, is approximately 25 minutes for a 3,600 points dataset, as demonstrated with the first blind sample, which was also the largest. This showcases the efficiency and practicality of this methodology, highlighting its rapid execution and suitability for in situ analysis. Such speed and effectiveness make it a valuable tool for real-time data collection and analysis in various scientific and industrial applications, allowing for timely and accurate decision-making.

## 4 Concluding remarks

This manuscript introduces an algorithm designed for the identification and discrimination of mineral types and their spatial distribution using Raman spectroscopy imaging. The approach presented applies an unsupervised clustering model supported by a processing pipeline designed to increase robustness, including baseline removal, Gaussian filtering, and data normalization. Intensity values for relevant mineral bands, chosen according to a mineral database, are extracted as input features of the clustering model, enabling the algorithm to effectively discriminate and spatially map mineral regions.

Indeed, the results for both training and blind test samples indicate a good correlation between the expected mineral type and model outcome, demonstrating both robustness and generalization capabilities. Furthermore, in order to enhance interpretability, visual tools were introduced to simplify mineral-cluster label assignment - allowing for an informed, interactive, and interpretable training stage - and results visualization - providing informative visual metrics to help the decision on the user side.

In terms of limitations, it is important to note that the success of this approach strongly depends on the complexity of the samples and the degree of distinctiveness of the mineral composition to be discriminated. The results suggest that distinguishing minerals with relevant bands that are mutually exclusive is clearly possible and easily automatized in robust manners. Nonetheless, the discrimination may become more challenging when dealing with minerals with similar Raman signatures, or in samples with a larger number of mineral types, for which mutually exclusive bands may be harder to find. In addition, the dimension of the spot size also limits the differentiation, in particular in the presence of fine grains. In such scenarios, future work may explore the extraction of a larger number of relevant features or even the fusion with other spectral techniques of higher throughput (e.g. hyperspectral reflectance imaging).

Along with these future improvements, the findings enclosed in the manuscript set the stage for future technological applications that may benefit and exploit this methodology. In particular, we foresee the possible development of a rapid and reliable tool for autonomous operation in mining sites during exploration and exploitation stages, having the potential to enhance safety in hazardous environments, save time and resources, and reduce environmental impact.

## CRediT authorship contribution statement

**Diana Guimarães:** Data analysis, Methodology, Writing and Review. **Catarina Monteiro:** Data acquisition, Methodology, Software, Writing and Review. **Joana Teixeira:** Algorithm development and Review. **Tomás Lopes:** Data analysis and Review. **Diana Capela:** Data interpretation and Review. **Filipa Dias:** Data acquisition and Review. **Alexandre Lima:** Data validation and Review. **Pedro A.S. Jorge:** Conception of the work, Writing - Review and Editing. **Nuno A. Silva:** Writing, Conceptualization of this study, Methodology, Software, Project administration.

## Declaration of Competing Interest

The authors declare the following financial interests/personal relationships which may be considered as potential competing interests: Diana Guimaraes reports financial support was provided by Instituto de Apoio às Pequenas e Médias Empresas e à Inovação. Diana Capela reports financial support was provided by Instituto de Apoio às Pequenas e Médias Empresas. Diana Guimaraes reports financial support and article publishing charges were provided by Foundation for Science and Technology. If there are other authors, they declare that they have no known competing financial interests or personal relationships that could have appeared to influence the work reported in this paper.

## Data Availability

The data and code used in the production of this manuscript can be made available upon reasonable request.
